# Dynamic Light Scattering Microrheology of Phase-Separated Poly(vinyl) Alcohol–Phytagel Blends

**DOI:** 10.3390/polym16202875

**Published:** 2024-10-11

**Authors:** Richa Ghosh, Sarah A. Bentil, Jaime J. Juárez

**Affiliations:** 1 Department of Mechanical Engineering, Iowa State University, Ames, IA 50011, USA; rg20@iastate.edu; 2 Center for Multiphase Flow Research and Education, Iowa State University, Ames, IA 50011, USA

**Keywords:** gellan, ergodic/non-ergodic, phase separation, microrheology, viscoelasticity, dynamic light scattering, hydrogel, heterogeneous medium

## Abstract

In this investigation, we explored the microrheological characteristics of dilute hydrogels composed exclusively of Poly(vinyl) alcohol (PVA), Phytagel (PHY), and a blend of the two in varying concentrations. Each of these polymers has established applications in the biomedical field, such as drug delivery and lens drops. This study involved varying the sample concentrations from 0.15% to 0.3% (*w*/*w*) to assess how the concentration influenced the observed rheological response. Two probe sizes were employed to examine the impact of the size and verify the continuity hypothesis. The use of two polymer blends revealed their immiscibility and tendency to undergo phase separation, as supported by the existing literature. Exploring the microrheological structure is essential for a comprehensive understanding of the molecular scale. Dynamic light scattering (DLS) was chosen due to its wide frequency range and widespread availability. The selected dilute concentration range was hypothesized to fall within the transition from an ergodic to a non-ergodic medium. Properly identifying the sample’s nature during an analysis—whether it is ergodic or not—is critical, as highlighted in the literature. The obtained results clearly demonstrate an overlap in the results for the storage (G’) and loss moduli (G″) for the different probe particle sizes, confirming the fulfillment of the continuum hypothesis.

## 1. Introduction

Dilute polymer solutions find extensive use in a variety of commercial and industrial applications, including contact lens solutions [1], cosmetics [2], lubrication [3], oil extraction from petroleum reservoirs [4], impurity absorption from water [5], emulsions [6], and drug delivery [7]. Hence, the characterization of the rheological properties of these polymers is critical to process engineering and quality assurance. The conventional approach is to utilize inline monitoring by siphoning a polymer solution from a process stream to characterize its properties by means of a viscometer [8,9]. Capillary and slit-die viscometers, for example, work by applying a known pressure or shear rate to extract the shear stress. This measurement yields insight into the complex viscosity of a polymer solution, from which rheological properties can be derived [10].

Although methods exist to rapidly assess the rheological properties of a variety of polymer solutions, dilute polymer solutions offer a unique challenge to viscometry. Typically, dilute polymer solutions exhibit low viscosities, which are difficult to acquire data from due to physical torque limitations in viscometry instrumentation [11]. Achieving high shear rates for dilute polymer solutions in capillary viscometers is also a challenge due to the onset of turbulence above shear rates of ~600 Hz [12]. Viscometry measurements are further complicated when only small volumes of the solution are available for measurement [13]. This issue frequently occurs in biological samples (e.g., cerebrospinal fluid), where the availability of large sample quantities is sometimes limited.

Various optical techniques have been developed to overcome the challenges posed by measuring low-viscosity fluids. A typical approach relies on dispersing small particles (~0.02% by volume) in the sample and then using a video camera to record the trajectories [14,15] or rotation [16] of these particles in solution. These data allow for the determination of the particle diffusion coefficient, and by extension, the solution viscosity through the calculation of parameters such as the mean-square displacement or cross-correlation function. Another approach to the optical interrogation of low-viscosity fluids relies on the optical trapping of particles [17,18,19]. This approach relies on video tracking or quadrant-positioning detection to measure the positions of trapped particles. A statistical analysis is then applied to the collected data to extract the dynamics of particle motion and infer the underlying system properties (e.g., viscosity, trap stiffness) [20,21].

Dynamic light scattering (DLS) is another optical technique that has found extensive use in the measurement of sample viscosity. Early studies that used DLS as a viscometry technique accomplished this measurement by determining the diffusion coefficient of a probe particle in a pure solvent and normalizing the result by the diffusion coefficient measured in a polymer solution [22]. This approach yields the relative viscosity of the sample and is a simple method by which to determine fluid viscosity. However, a comprehensive analysis [23,24] of DLS data showed that the intensity autocorrelation function obtained from DLS is related to the mean-square displacement, from which viscoelastic properties can be determined.

The recognition that DLS data can yield insight into viscoelastic properties created opportunities to study a wide range of polymer properties. For example, characterizing the decay rate of the intensity autocorrelation as a function of the scattering angle serves as a measure of the polymer elastic compression modulus [25]. DLS can be used to map the dynamics of polymers as a function of temperature and concentration, thus enabling the study of polymer phase behavior [26]. The intensity autocorrelation data obtained from DLS can be used to reconstruct the microrheological behavior of polymeric materials over a broad frequency spectrum (~6 decades) [27]. Examples of applied DLS-based microrheological analyses include the examination of polymer gelation [28], bonding kinetics [29], aging [30], and viscoelastic properties of both human tear drops and artificial tear droplets [31].

This paper presents the results of an experimental study of dilute poly(vinyl) alcohol (PVA) and phytagel (PHY) blends using DLS microrheology. Recently, PVA-PHY composites were proposed as tissue mimics due to their mechanical similarity to brain [32] and liver [33,34] tissues. However, knowledge of how the rheological properties of PVA-PHY affect the microscale, where cellular interactions are important, is limited. This experimental study examined PVA, PHY, and their blends in the range of 0.15 wt% to 0.3 wt%, where the experimental results indicate that phase separation occurred. The separation of the polymer phases will lead to anisotropic PVA-PHY composite properties that influence their mechanical similarity to biological tissues. Our DLS results indicate that the phase transition was characterized by a transition from ergodic to non-ergodic behavior. Thus, the novelty of this work lay in the use of DLS microrheology to characterize the dilute PVA-PHY solutions, which highlighted the phase separation of the polymer blend at lower concentrations. The results from this work can aid in the formulation of new types of tissue mimics using polymer composites [31], where phase separation is carefully controlled at high concentrations or for the development of bio-inks [32,33,35] in the dilute range. Moreover, many researchers used PHY/PVA blends to formulate implants [36] and tissues, both of which require the product to be stable. Our research on the lower concentration range highlights the need to improve the stability of polymer blends.

## 2. Materials and Methods

### 2.1. Sample Preparation

The PVA (Acros Organics, Waltham, MA, USA, catalog number: 183120010) used in the experiments was 98.0–98.8% hydrolyzed, with a molecular weight that ranged from 146,000 to 186,000. The PHY (Sigma-Aldrich, St. Louis, MO, USA, product number: P8169) had a molecular weight of 1000 kg/mol. For this study, we selected polystyrene particles with nominal diameters of 0.5 μm and 1 μm to serve as probes for the DLS experiments. The particles were sourced from Polysciences (cat # 07307-15, 07310-15). The deionized (DI) water used in the sample preparation was filtered three times with a 0.2 μm Whatman Uniflo Syringe Filter (lot no: 230416-419-A) to remove dust and other suspended particles.

The polymer solutions were prepared using the procedures outlined by Forte et al. [32]. These polymer solutions were dilute in nature and comprised mainly of water. The hydrogels were formed mainly by physical entanglement, as observed using a microscope. The PHY powder was added to the DI water in a beaker and gradually heated for 30 min to reach 90 °C while stirring with a stir bar rotating at 500 rpm. Aluminum foil was placed on top of the beaker to prevent water evaporation and maintain uniform heating. The procedure for preparing the PVA solutions was like the PHY preparation, except that the PVA solutions were heated for 1 h, and thereafter, was frozen at −18 °C for 15 h. The preparation procedure followed 1 freeze–thaw cycle. The blends were formulated by individually creating the respective PHY and PVA solutions, except in this instance, both the PHY and PVA were heated for 1 h at 90 °C at 500 rpm. Thereafter, the polymeric PHY solution and PVA solution were mixed together in a 1:1 ratio. The mixture was heated for 30 min while being stirred at 300 rpm at 70 °C. The solution of blended polymers was then allowed to cool once the mixing procedure was complete. No external crosslinker was added. The procedure for formulating the blends was taken from the work undertaken by Kainz et al. [37]. Polystyrene probe particles (0.1% *w*/*v*) were pipetted and dispersed in the polymer solutions. A vortex mixture was used to ensure a uniform dispersion in the medium, and thereafter, the sample was frozen for 16 h at −20 °C. The sample was thawed at room temperature (24 °C) for 8 h before it was ready for experimentation.

### 2.2. Dynamic Light Scattering Experiments

**Figure 1:**
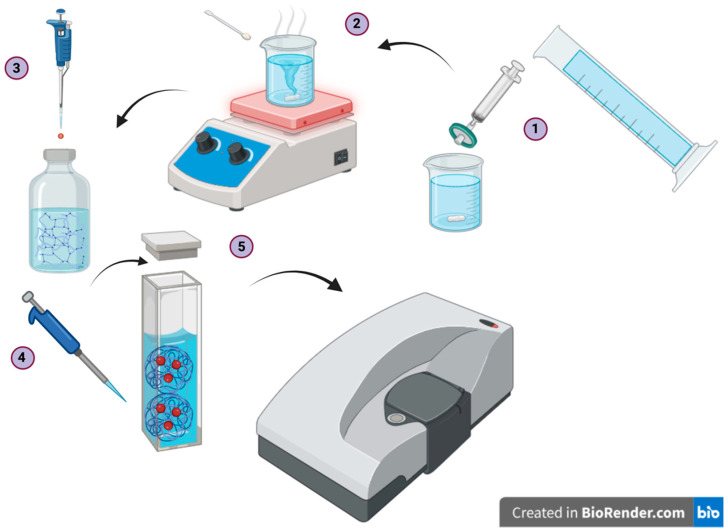
Experimental procedure for the sample preparation and dynamic light scattering (DLS) experiments aimed at extracting the rheological parameters. Latex probe particles of sizes ∅ 0.51 μm and ∅ 1.01 μm were used. The experimental procedure consisted of five key steps: (1) filtration of the deionized water, (2) preparation of the polymer solution batch, (3) pipetting of the probes into the sample, (4) pipetting the sample into the cuvette, and (5) conducting the dynamic light scattering experiment using Zetasizer.

Dynamic light scattering measurements were performed after a freeze–thaw of the collected samples (PVA) and different blends of PVA-PHY. Figure 1 shows the process for the experiments described here. DLS experiments were performed at a controlled temperature of 25 °C using the Malvern Zetasizer Nano ZS series instrument, Malvern, UK, with a helium–neon (HeNe) laser that operated at a wavelength of 632 nm focused on the sample in a disposable 4.5 mL ultraviolet (UV) cuvette (Fisherbrand, Waltham, MA, USA, cat. no. 14955130). The scattering was detected at a backscattering angle of 180° to reduce the noise. The sizes of the probe particles used, in this case (∅ 0.51 μm, ∅ 1.01 μm) latex particles, were chosen to examine the degree of overlap in the measurement of the samples’ viscous and storage moduli. If the observed data overlapped, then this suggested that the effect of the particle size on the measurements was negligible. Table 1 provides information on the polymer blends examined in this work. All polymer concentrations presented in Table 1 are reported in units of % (*w*/*w*), unless otherwise stated. The density of the polystyrene probe particle (1.05 g/cm^3^) was close to the density of the hydrogel, which predominantly consisted of water (~1 g/cm^3^). This helped to minimize the effect of sedimentation on our results. Our experiments primarily focused on two blend combinations: Blend 1, which consisted of PVA 0.15% (*w*/*w*) + PHY 0.15–0.30% (*w*/*w*), and Blend 2, which consisted of PVA 0.225% (*w*/*w*) + PHY 0.15–0.30% (*w*/*w*). The hydrogels encompassed dilute solutions of PHY, PVA, Blend 1, and Blend 2.

While analyzing the DLS experimental results, it was crucial to take into consideration the nature of the sample being studied. In most cases, a gel can be safely considered non-ergodic due to limited Brownian motion about a set of fixed positions dictated by the mesh formed in the polymer blend. However, as we dealt with very low concentrations, determining the nature of the sample was achieved by studying the overlap in the measured intensity autocorrelation function (IACF). If the repeated time-averaged measurements lead to a non-overlapping result at correlation time (time interval between data points) τ = 0, and at τ→∞ the normalized IACF decorrelates to 0, then the sample can safely be considered non-ergodic [38,39]. In this work, DLS measurements were performed at 20 different positions using a 100 μm step size. If all 20 measurements overlapped, then the sample was considered ergodic. This was implemented by following the procedures of Cai et al. [27]. However, if any of the measurements deviated in a manner like what is described above, then the sample was considered non-ergodic.

The microscopy images of the polymer blend were captured using an OLYMPUS IX51 Microscope with 20× objective lens. The image was scaled using a 10 μm scale bar. As discussed in the Section 4, the images clearly show polymer entanglement that was not uniform throughout and there were signs of phase separation.

## 3. Theory

DLS measurements yield intensity data as a function of time. Extracting the rheological behavior of the sample begins with obtaining the normalized time-averaged intensity autocorrelation function (IACF):

(1) $$g_{2}\left(q,\tau \right)=\frac{\langle I\left(q,t\right)I(q,t+\tau \rangle }{I{\left(q,t\right)}^{2}}$$

where $q=4\pi \;n\;sin\left(\theta /2\right)/\lambda$ is the scattering wave vector, *n* is the index of refraction, *q* is the scattering angle, *λ* = 633 nm is the wavelength of the laser source, *τ* is the time interval between data points, *I* is the intensity, and *t* is the time. The IACF is the autocorrelation of the intensity measured in DLS experiments normalized by the square of the intensity at time *t*.

In fluid-like media, the particles are free to diffuse throughout, allowing for quick samples of many configurations [39]. Under these conditions, the medium is considered ergodic and the Siegert relation may be used to connect the IACF parameter $g_{2}$ to the parameter *g*_1,_ known as the normalized field autocorrelation function (FACF). The IACF and FACF are related as follows:

(2) $$g_{2}\left(q,\tau \right)=1+\beta^{2}{\left|g_{1}\left(q,\tau \right)\right|}^{2}$$

where β is the coherence factor, which accounts for the number of speckles or coherence area detected. In ideal conditions, β≤1. However, the assumption that particles diffuse quickly and display many configurations does not hold in gel-like media. In this situation, the probe is confined by the viscoelastic response of the medium [40], giving the appearance of being fixed in place. Hence, gel-like media exhibits a non-ergodic response. The FACF may then be evaluated as follows [41,42]:

(3) $$\begin{array}{c} g_{1}\left(q,\tau \right)=\frac{1}{Y}\left[\left(Y-1\right)+\sqrt{g_{2}\left(q,\tau \right)-\sigma_{1}^{2}}\right]\\ \sigma_{1}^{2}=\langle I^{2}\left(q,t\right)/I{\left(q,t\right)}^{2}-1\rangle \\ Y={\langle I\rangle }_{e}/{\langle I\rangle }_{t} \end{array}$$

where ${\langle I\rangle }_{e}$ is the total intensity averaged over an ensemble of different volumes and ${\langle I\rangle }_{t}$ is the time-averaged total scattered intensity for the volume being studied.

For the reader’s understanding, the time-averaged intensity correlation function (ICF) is obtained by averaging over an appropriate sub-ensemble of the sample. The ensemble-averaged intensity correlation function (ICF) is obtained by ensemble-averaging properties of the medium. In the case of an ergodic medium, the time-averaged intensity correlation function equals the ensemble-averaged intensity correlation function. The time-averaged correlation functions overlap with each other at τ=0. For a non-ergodic medium, the time average and ensemble average are not equal and the time-averaged intensity correlation functions do not overlap with each other at τ=0.

The evaluation of rheological properties is achieved using the DLS microrheology (DLSμR) protocol of Cai et al. [27]. In this approach, the IACF is smoothed using a fit of the form

(4) $$g_{2}\left(q,\tau \right)=1+x_{o}exp\left(-\alpha t^{\beta }\right)$$

where *x_o_*, α, and β are fitting parameters. The FACF can be determined by applying Equation (4) to Equation (2) or Equation (3), depending on the ergodic nature of the material. The FACF is directly correlated to the dynamics of the particles dispersed in the medium [43]:

(5) $$g_{1}\left(q,\tau \right)=exp\left(-q^{2}\langle r^{2}\left(t\right)\rangle /6\right)$$

where $\langle r^{2}\left(t\right)\rangle$ is the mean-square displacement (MSD) measured by the DLS instrument. The MSD is then used to evaluate the rheological properties by using a combination of two approaches, as suggested by Cai et al. [27]. We summarize these approaches here. At intermediate frequencies, the Laplace transform of the MSD is evaluated numerically to calculate the complex modulus $\tilde{G}$:

(6) $$\tilde{G}\left(s\right)=\frac{k_{B}T}{\pi asL\left\{\langle r^{2}\left(t\right)\rangle \right\}}$$

where *k_B_* is Boltzmann’s constant, *T* is the sample temperature, *a* is the probe radius, *s* = *iω* is the complex frequency domain parameter, and L is the Laplace transform. The real component of Equation (6) represents the storage modulus (G’), while the imaginary component represents the loss modulus (G″). The frequency responses of the material at the upper and lower ranges are evaluated using a power law approach [44]. In this approach, the complex modulus $\tilde{G}\left(s\right)$ is evaluated by numerically converting the MSD to Fourier space using the generalized Stokes–Einstein (GSE) equation:

(7) $$\tilde{G}\left(\omega \right)=\frac{k_{B}T}{\pi a\left(i\omega \right)\;F_{u}\left\{\langle r^{2}\left(t\right)\rangle \right\}}$$

Viscoelastic properties obtained by using the GSE equation are applicable only when the continuum assumption is valid, which is that the length scale of the probe particle should scale to that of the characteristic length scale of the polymer mesh. The Fourier transform is determined by assuming that the MSD follows a power law model:

$$i\omega F_{u}\left\{\langle r^{2}\left(t\right)\rangle \right\}\approx \langle r^{2}\left(1/\omega \right)\rangle \Gamma \left[1+\alpha \left(\omega \right)\right]i^{-\alpha \left(\omega \right)}$$

where Γ is the gamma function and α is the power law exponent. Hence, combining Equations (6) and (7) allows for the determination of rheological properties over the full range of experimentally sampled observations.

## 4. Results

**Figure 2:**
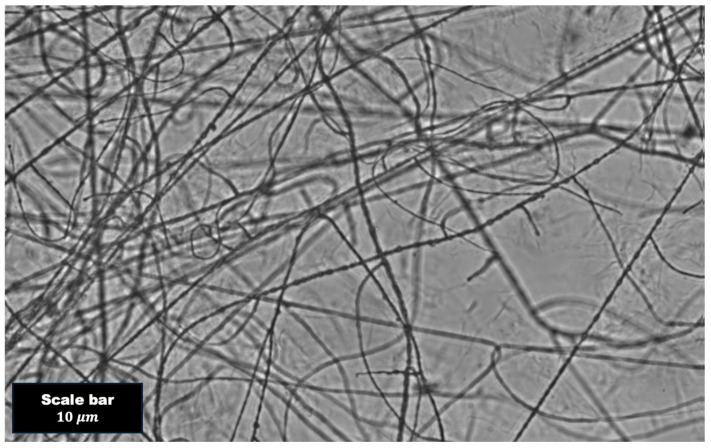
Microscopic image of the polymer structure (subset of Blend 1) comprising PVA 0.15 wt%-PHY 0.30 wt%. It clearly demonstrates that the polymers were physically overlapped.

As a control for our polymer-blending experiments, DLS experiments were performed in PHY and PVA at concentrations of 0.15% (*w*/*w*), 0.225% (*w*/*w*), and 0.3% (*w*/*w*). We further performed experiments on Blend 1 and Blend 2, as discussed in a later section. Our results indicate that some samples exhibited ergodicity, while other samples were non-ergodic. The distinction between these properties was likely the result of polymer phase separation. This was backed up by capturing microscopy images of the polymer blend (e.g., Figure 2). Figure 2 shows interconnected polymer strands that fell out of the solution. Although PVA blended with other polymers (e.g., PHY) have been reported as successfully forming a hydrogel with an intertwined network [32], the results we discuss below indicate that the PVA blends were susceptible to phase separation. This was also observed in other research that examined PVA composite materials [45].

**Figure 3:**
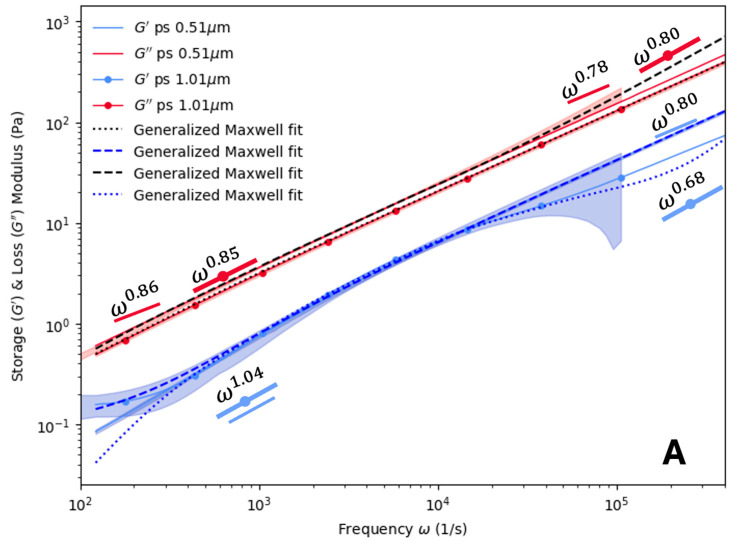

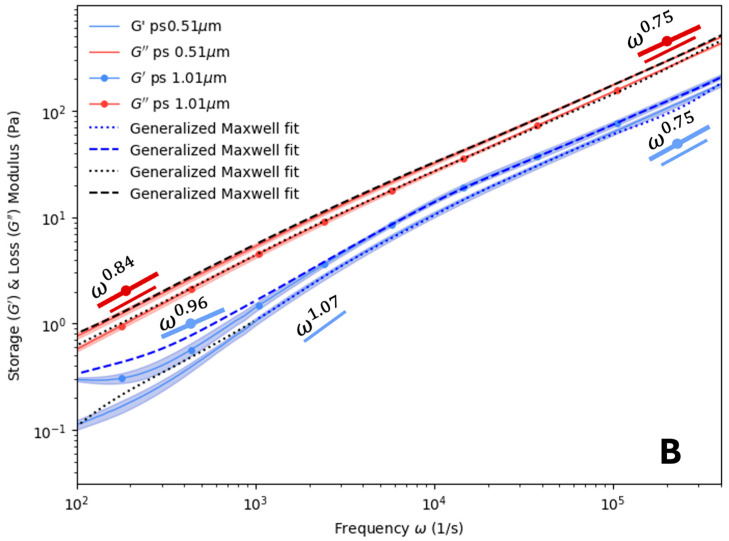
Storage and loss modulus of PHY at varied concentrations: (**A**) 0.15% (*w*/*w*) and (**B**) 0.30% (*w*/*w*) for two probe sizes: ∅ 0.51 μm and ∅ 1.01 μm. Opaque regions indicate one standard deviation from the mean, *n* = 3. Maxwell modeling was used to fit the curves. Scaling (α) values for ∅ 0.51 μm and ∅ 1.01 μm latex probe particles are also plotted.

The PHY results are presented in Figure 3. Two different probe diameters (0.51 μm and 1.01 μm) were utilized as a check to ensure that the produced results were consistent. Aside from some deviations at high or low frequencies, the storage and loss modulus overlap for all PHY concentrations examined in Figure 3. The overlapping for both probe types indicates that a fluid with similar properties was sampled. At these concentrations, the measurements were independent of the probe size. Additionally, G′ was less than G″ within the frequency range examined. This suggests that the PHY solutions studied could be characterized as viscous, rather than elastic, over the observed frequency range. For the value of α at low frequencies, values of 0.96 to 1.07 were found for G′ and 0.84 to 0.86 for G″. At higher frequencies, the values of α varied from 0.68 to 0.80 for G′ and 0.75 to 0.8 for G″. At high frequencies, the values of α for G″ appear to fall in between what one would expect for a Newtonian fluid (~ω) and a dilute or semi-dilute polymer solution following the Zimm model ($\sim \omega^{2/3}$) [46]. There did not appear to be any variation based on the probe diameter or concentration. No crossover between the storage and loss moduli were observed in any of the subplots of Figure 3 for the PHY sample.

**Figure 4:**
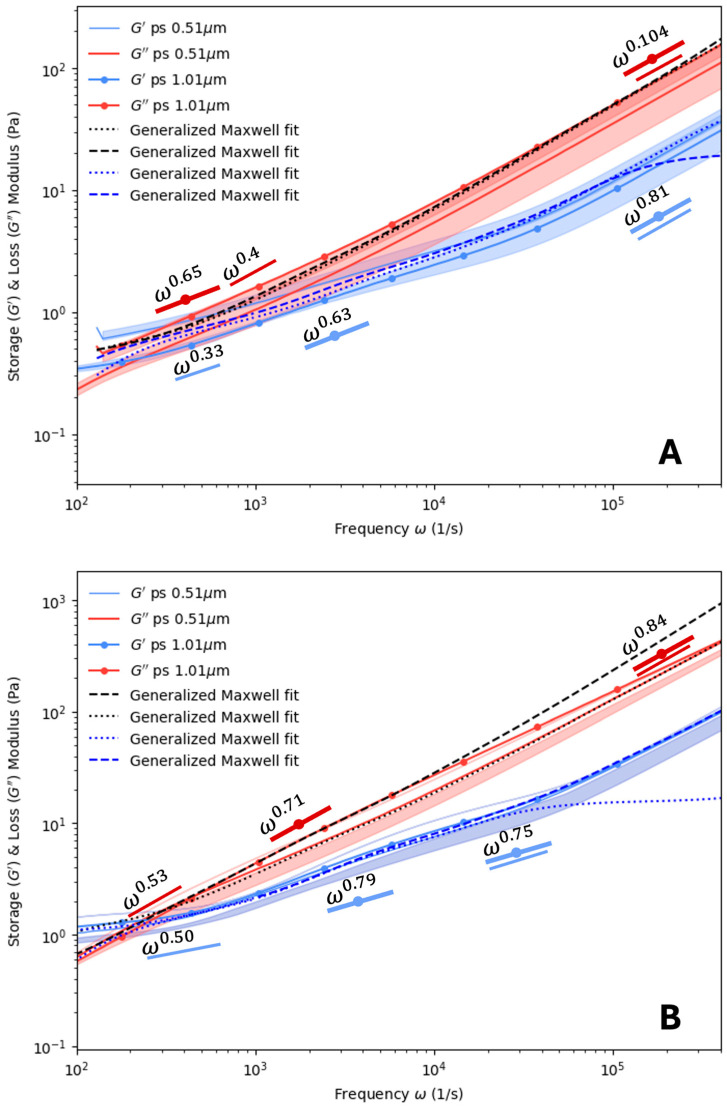
Storage and loss modulus of poly(vinyl) alcohol at varied concentrations: (**A**) 0.15% (*w*/*w*) and (**B**) 0.30% (*w*/*w*) for two probe sizes: ∅ 0.51 μm and ∅ 1.01 μm. Opaque regions indicate one standard deviation from the mean, *n* = 3. Maxwell modeling was used to fit the curves. Scaling (α) values for ∅ 0.51 μm and ∅ 1.01 μm latex probe particles are also plotted.

Figure 4 shows the DLS microrheology data for PVA at 0.15% (*w*/*w*) and 0.3% (*w*/*w*). An examination of α for low frequencies showed that the power factor had values of 0.33 to 0.79 for G′ and 0.4 to 0.71 for G″. PVA was noted to exhibit a wider degree of variability in α than the PHY samples. It appears that α increased with the particle diameter and polymer concentration. Furthermore, it appears that PVA at these concentrations and at low frequencies resembled a concentrated polymer melt consistent with the Rouse model (~$\omega^{1/2}$) [46]. This suggests that the material had elastic characteristics, which crossed over to viscous behavior at frequencies below 1 kHz. At higher frequencies, the values of α ranged from 0.75 to 0.81 for G′ and 0.84 to 1.04 for G″. Hence, once the frequency exceeded the crossover point, the PVA transitioned from a concentrated polymer melt into a semi-dilute polymer solution. From Figure 4A,B, the point of crossover for the storage and loss modulus curves was ~2.7 kHz for a concentration of 0.15% (*w*/*w*). With the increase in concentration to 0.225% (*w*/*w*), the crossover point shifted to ~347 Hz, and for 0.30% (*w*/*w*), the crossover point shifted further back to ~322 Hz.

**Figure 5:**
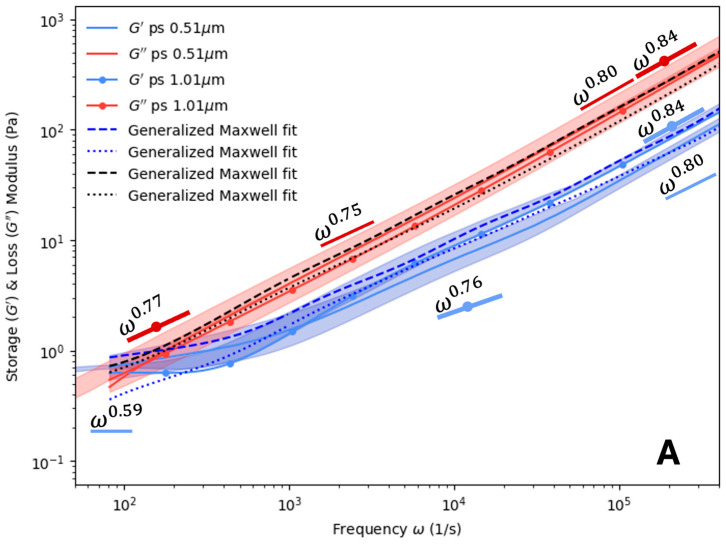

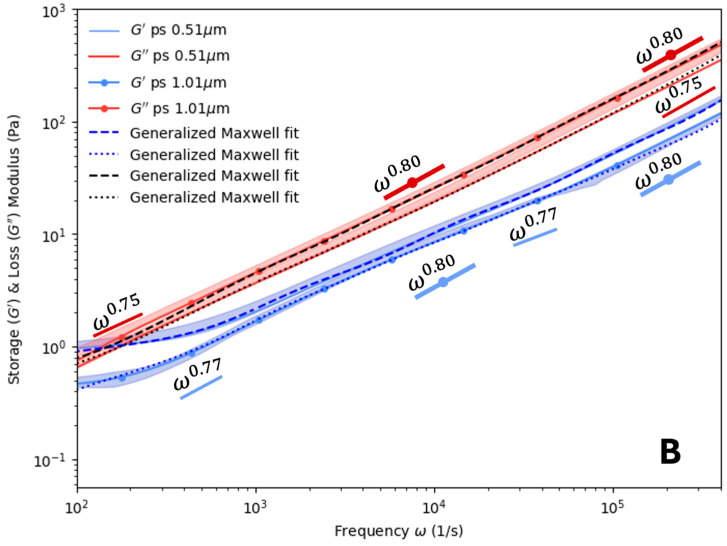
Storage and loss modulus analysis in the non-ergodic/ergodic medium Blend 1 with Polystyrene probes: impacts of particle size and polymer immiscibility. (**A**) PVA 0.15, PHY 0.15 and (**B**) PVA 0.15, PHY 0.30 using two probe particles: ∅ 0.51 μm and ∅ 1.01 μm. The discrepancy at lower frequencies was due to the polymer immiscibility. A fitting method that combined a Laplace transform and power law resulted in accuracy limitations. Opaque regions indicate one standard deviation from the mean, *n* = 3. Maxwell modeling was used to fit the curves. Scaling (α) values for ∅ 0.51 μm and ∅ 1.01 μm latex probe particles are also plotted.

**Figure 6:**
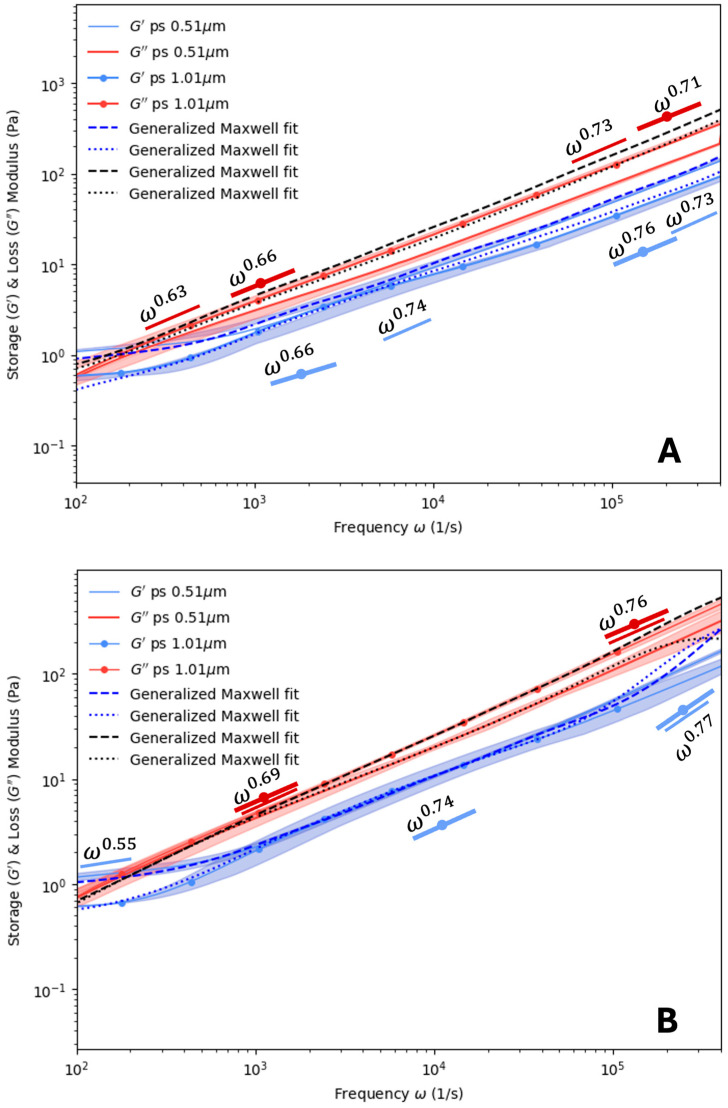
Storage and loss modulus modulus analysis in non-ergodic/ergodic medium Blend 2 with polystyrene probes: impacts of particle size and polymer immiscibility. (**A**) PVA 0.225, PHY 0.15 and (**B**) PVA 0.225, PHY 0.30. Opaque regions indicate one standard deviation from the mean, *n* = 3. Maxwell modeling was used to fit the curves. Scaling (α) values for ∅ 0.51 μm and ∅ 1.01 μm latex probe particles are also plotted.

With the baseline rheological behavior established for PHY and PVA, we turned our attention to blending these materials together and using DLS to measure the rheological responses of the dispersed probes. The results are presented in Figure 5 through Figure 6. Figure 5 shows the results for Blend 1 (see Table 1), which consisted of a fixed concentration of PVA (0.15% *w*/*w*) blended with varying concentrations of PHY (0.15% *w*/*w* to 0.3% *w*/*w*). For a concentration of 0.3% *w*/*w* of PVA (Figure 5B), the value of α did not vary significantly. In Figure 5A,B, there appears to be a crossover point near a frequency of ~100 Hz, an artifact that did not appear in any of the PHY data. This indicates that PVA influenced the rheology by making these samples appear more elastic than the PHY (Figure 3). The results also show that there was a considerable overlap in the data points at frequencies above ~1 kHz for both probe sizes, indicating that the probe size did not influence the data in this range. However, below this range, there was a deviation in the measured data. This indicates that the measured elastic response was influenced by the probe size. From Figure 5A,B, the crossover point was ~60 Hz for the Blend 1 concentrations of PVA 0.15, PHY 0.15. This cross-over shifted to the right as the concentration of the PHY increased, with the crossover reported at ~67 Hz for PVA 0.15, PHY 0.225 and the crossover reported at ~183 Hz for PVA 0.15, PHY 0.30.

Figure 6 shows the results for Blend 2, where the PVA concentration was fixed at 0.225% *w*/*w* while the PHY concentration was varied. The crossover between G′ and G″ that started appearing in Figure 5 became more pronounced, with elastic tails that extended below 100 Hz in some cases. Below this frequency range, the elastic response dominated the viscous response of the fluid. At high frequencies (ω→10^6^ Hz), the values of α for both G′ and G″ varied from 0.71 to 0.77. These values were generally lower than the 0.75 to 0.84 range observed in Figure 4. Additionally, there was significant deviation in the measurements of the two probe sizes at frequencies below ~1 kHz. This indicates that the elastic response of the fluid significantly influenced the dynamics of the probes. The crossover frequency for PVA 0.225, PHY 0.15 was ~55 Hz; for PVA 0.225, PHY 0.225, it was 91.32 Hz; and for PVA 0.225, PHY 0.30, it was ~ 80 Hz.

In Figure 5, for the most part, the loss and storage moduli measured by the two probe sizes appear to overlap. As the hydrogel was formed by a very dilute concentration, it was expected that the loss modulus would be of a higher magnitude than that of the storage modulus, as can be seen. A similar observation was noted for Blend 2, which consisted of PVA 0.225 (*w*/*w*%) mixed with varied concentrations of PVA 0.15–0.30% *w*/*w*% in Figure 6.

The experimental results in Figure 4, Figure 5 and Figure 6 were validated with the results obtained using the generalized Maxwell model. In our work, RepTate rheology software (version 1.3.3) [37] was used to model our system. The generalized Maxwell model was used to fit the experimental data. The actual relaxation of the polymer blend relaxed in the spectrum time frame, which was the specific duration chosen over which data was collected to generate the spectral analysis. Care was taken to choose a time frame that allowed for good frequency resolution to detect transient phenomena within the signal. The loss and the storage modulus in such a case can be fitted using the generalized Maxwell model expressed as

(8) $$G^{\prime }\left(\omega \right)=\sum_{i=n}^{N}\frac{G_{i}{\left(\omega \lambda_{i}\right)}^{2}}{1+{\left(\omega \lambda_{i}\right)}^{2}}$$

(9) $$G^{\prime \prime }\left(\omega \right)=\sum_{i=n}^{N}\frac{G_{i}\left(\omega \lambda_{i}\right)}{1+{\left(\omega \lambda_{i}\right)}^{2}}$$

The parameters for the generalized Maxwell model are given in the following tables: Tables S2–S5 in the Supplementary Materials. The fitting procedure was performed using at least 72 data points, with a maximum of 24 terms for Equations (8) and (9). This ensured that there was no overfitting of the experimental data using the generalized Maxwell model. The scaling analysis complied with the power law exponent in the Fourier domain. Using the generalized Maxwell model, the characteristic range of the relaxation time of PHY was observed to lie in the range of (2.67 × 10^−8^ s, 0.0473602 s); for PVA, it was found to lie in the range of (1.44 × 10^−8^ s, 0.155331 s); for Blend 1, which consisted of PVA 0.15 + PHY 0.15–0.30% (*w*/*w*), in the range (4.3 × 10^−8^ s, 0.0453851 s); and for Blend 2, which consisted of PVA 0.225 + PHY 0.15–0.30% (*w*/*w*), in the range of (1 × 10^−8^ s, 4.56868 s).

**Figure 7:**
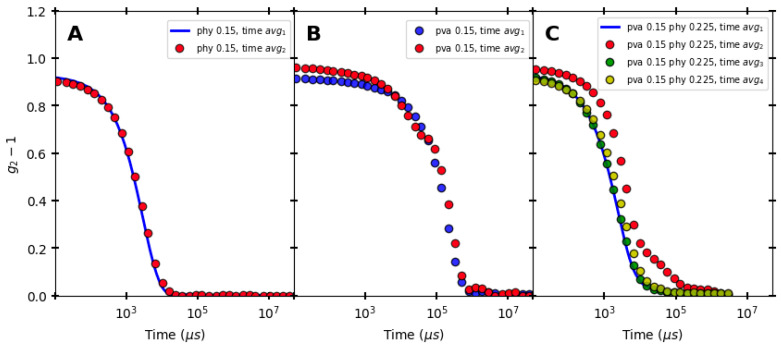
Auto-correlation function (ACF) for (**A**) PHY, (**B**) PVA, and (**C**) Blend 1 at T = 25 °C. The ensemble averaging was conducted across various positions, as outlined in the Supplementary Materials Table S1. In (**A**), the ACF for PHY overlapped at small time scales, indicating an ergodic medium. In (**B**), the ACF for Poly(vinyl) alcohol did not overlap at small time scales, indicating a non-ergodic medium. In (**C**), some ACFs for Blend 1 overlapped at small time scales, but time average 2 did indicate a transition between ergodic and non-ergodic states. This trend was also observed in repeated experiments with Blend 2. For the analysis, both Blend 1 and Blend 2 were considered non-ergodic media.

Figure 7 shows overlapping of time-averaged intensity autocorrelation function for PHY, PVA, and Blend 1. A mono-decay was observed. As can be seen from Figure 7A, the time-averaged ACFs for PHY were reproduced nicely at τ→0; hence, it was concluded that the polymer solution formed from PHY was ergodic in nature. Taking this into consideration, the analysis for PHY used Siegert’s relation (Equation (2)). In Figure 7B, the time-averaged ACFs for PVA did not overlap well at τ→0. In Figure 7C, four out of the five samples in the case of Blend 1 overlapped at τ→0. In case of the reading for (PVA 0.15-PHY 0.225, time avg_2_) at τ→0, the time average did not overlap, except for when τ→∞, which is a characteristic of a non-ergodic medium. As mentioned previously, for an intermediate non-ergodic medium, the ensemble-averaged normalized ACF generally decays to a constant non-zero value [39], but in our case, even for non-ergodic media (PVA and Blend 1), the normalized ACF was found to decorrelate to 0 instead of a non-zero constant value. Since our concentration lay in the intermittent transitional range of ergodicity and non-ergodicity, and since it was found that the sample underwent a phase separation, for the same sample concentration, it showcased both ergodicity (overlapping of time-averaged ACF at τ→0) and non-ergodicity (non-overlapping time-averaged ACF at τ→0) characteristics.

## 5. Conclusions

In this work, we utilized dynamic light scattering to measure the rheological properties of dilute PHY and PVA blends. These experiments demonstrated that the polymer blends exhibited properties that were distinct from dilute solutions of PHY and PVA prepared alone. Our findings for the dilute range appear to indicate that there was a transition from ergodic to non-ergodic behavior in these polymer solutions. This transition appears to be characterized by the phase separation of PVA and PHY when blended together. This observation has implications for the preparation of blends using higher concentrations of these polymers, which could continue to exhibit non-ergodic behavior. One goal of future work will be to examine the behavior of higher concentrations and characterize the impact that non-ergodicity has on rheological properties. Furthermore, this work is relevant for researchers studying the rheological properties of composite hydrogels, which have diverse applications, such as tissue mimics (e.g., brain phantoms), lens drops, drug delivery, and oil extraction from wells. It is valuable for those dealing with complex heterogeneous media, especially when working with a mixture of ergodic and non-ergodic media. DLS, especially being a cost-effective experimental tool, can be used in conjunction with other measurement tools, such as a conventional rheometer, optical tweezers, and/or atomic force microscopy, to conduct multispectral analysis. Its low sample requirement and the automation sampling edge can be a lucrative prospect for the rapid inline monitoring of the viscosity and rheology of complex fluids with little human intervention. Using these advantages of the system, it will be worthwhile to probe various complex fluids across multiple spectra and concentration ranges in future work.

## Figures and Tables

**Table 1:** Blend combinations used in this experiment. All reported values are in units of % (*w*/*w*).

		PHY	0.15	0.225	0.30
	PVA				
Blend 1	0.15		(0.15, 0.15)	(0.15, 0.225)	(0.15, 0.30)
Blend 2	0.225		(0.225, 0.15)	(0.225, 0.225)	(0.225, 0.30)
Blend 3	0.30		(0.30, 0.15)	(0.30, 0.225)	(0.30, 0.30)

## Data Availability

The original data presented in this study are openly available in the Article/Supplementary Materials and from the authors upon request.

## Supplementary Materials

The following supporting information can be downloaded from https://www.mdpi.com/article/10.3390/polym16202875/s1, Figure S1, The elastic & viscous moduli read for PHY 0.03wt% with latex particles of varying size. Limitations of Detection Method at lower frequency limits attributed to lower detection limit of the method. Figure S2, The elastic & viscous moduli read for Blend 3 PVA 0.30, PHY 0.15 with latex particles of varying size. At the lower and upper frequency limits, the non-overlapping results may be due to phase separation of the medium. Figure S3, Confinement Effects in Poly(vinyl) alcohol and Phytagel: concentration-dependent mean square displacement analysis. Figure S4, The mean square displacement of blends having different size probe particles showcasing confinement, as expected. The higher the concentration, the more confinement can be seen from the graph, as expected. Figure S5, Microscopic Image of Sample (PVA 0.15, PHY 0.15% *w*/*w*) Captured with OLYMPUS IX51 Microscope. The polymer sample relates to physically entangled network. Figure S6, Microscopic Image of Sample (PVA 0.225, PHY 0.30% *w*/*w*) captured with OLYMPUS IX51. Microscope. Figure S7, Auto-correlation function of Blend 2. Experimental Measurements at T = 25 °C. Ensemble averaging conducted across various positions. Table S1, Experimental procedure for conducting experiments for non-ergodic medium. Table S2, Parameters for generalized Maxwell model for Phytagel of various concentrations with Polystyrene probe particles (ps) of size 0.51 μm and 1.01 μm embedded. Table S3, Parameters for generalized Maxwell model for Poly(vinyl) alcohol of various concentrations with Polystyrene probe particles (ps) of size 0.51 μm and 1.01 μm embedded. Table S4, Parameters for generalized Maxwell model for Blend 1: Poly(vinyl) alcohol 0.15% (*w*/*w*) and Phytagel 0.15–0.30% (*w*/*w*) of various concentrations with Polystyrene probe particles (ps) of size 0.51 μm and 1.01 μm embedded. Table S5, Parameters for generalized Maxwell model for Blend 2: Poly(vinyl) alcohol 0.225% (*w*/*w*) and Phytagel 0.15–0.30% (*w*/*w*) of various concentrations with Polystyrene probe particles (ps) of size 0.51 μm and 1.01 μm embedded. Table S6, Insights on the distribution of data for samples with repetition of 3, n = 3 at ω = 80000 Hz.
