# Supplementary material for: Dynamic Light Scattering Microrheology of Phase-Separated Poly(vinyl) Alcohol–Phytagel Blends
Source: Polymers (Basel). 2024 Oct 11;16(20):2875. doi: 10.3390/polym16202875 (PMC11510749; doi:10.3390/polym16202875)
Supplement: Supplementary file 1 [file polymers-16-02875-s001.zip › polymers-3237380-supplementary.pdf]

polymers

MDPI

Supplementary Materials

Dynamic Light Scattering Microrheology of Phase-Separated Poly(vinyl) Alcohol–Phytagel Blends

Richa Ghosh [1], Sarah A. Bentil [1,*] and Jaime J. Juárez [1,2,*]

[1] Department of Mechanical Engineering, Iowa State University, Ames, IA 50011, USA; rg20@iastate.edu
[2] Center for Multiphase Flow Research and Education, Iowa State University, Ames, IA 50011, USA
* Correspondence: sbentil@iastate.edu (S.A.B.); jjuarez@iastate.edu (J.J.J.); Tel.: +1-515-294-8528 (S.A.B.); +1-515-294-3298 (J.J.J.)

Table S1. Experimental procedure for conducting experiments for non-ergodic medium.

Step	Duration	Description
1	5 mins	Equilibration period for desired temperature
2	30 mins	Scattering Measurement (Optimum Position: 4.6 mm)
3	30 mins	Scattering Measurement (Optimum Position: 4.6 mm)
4	10 secs	Scattering Measurement (Optimum Position: 4.45 mm)
5	10 secs	Scattering Measurement (Optimum Position: 4.35 mm)
6	10 secs	Scattering Measurement (Optimum Position: 4.25 mm)
8	10 secs	Scattering Measurement (Optimum Position: 4.15 mm)
9	10 secs	Scattering Measurement (Optimum Position: 4.05 mm)
10	10 secs	Scattering Measurement (Optimum Position: 3.95 mm)
11	10 secs	Scattering Measurement (Optimum Position: 3.85 mm)
12	10 secs	Scattering Measurement (Optimum Position: 3.75 mm)
13	10 secs	Scattering Measurement (Optimum Position: 3.65 mm)
14	10 secs	Scattering Measurement (Optimum Position: 3.55 mm)
15	10 secs	Scattering Measurement (Optimum Position: 3.45 mm)
16	10 secs	Scattering Measurement (Optimum Position: 3.35 mm)
17	10 secs	Scattering Measurement (Optimum Position: 3.25 mm)
18	10 secs	Scattering Measurement (Optimum Position: 3.15 mm)
19	10 secs	Scattering Measurement (Optimum Position: 3.05 mm)
20	10 secs	Scattering Measurement (Optimum Position: 2.95 mm)
21	10 secs	Scattering Measurement (Optimum Position: 2.85 mm)
22	10 secs	Scattering Measurement (Optimum Position: 2.75 mm)
23	10 secs	Scattering Measurement (Optimum Position: 2.65 mm)
24	10 secs	Scattering Measurement (Optimum Position: 2.55 mm)

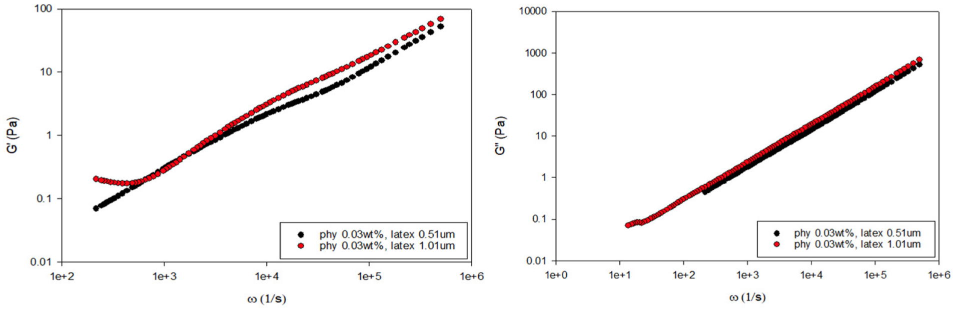

Figure S1. The elastic & viscous moduli read for PHY 0.03wt% with latex particles of varying size. Limitations of Detection Method at lower frequency limits attributed to lower detection limit of the method.

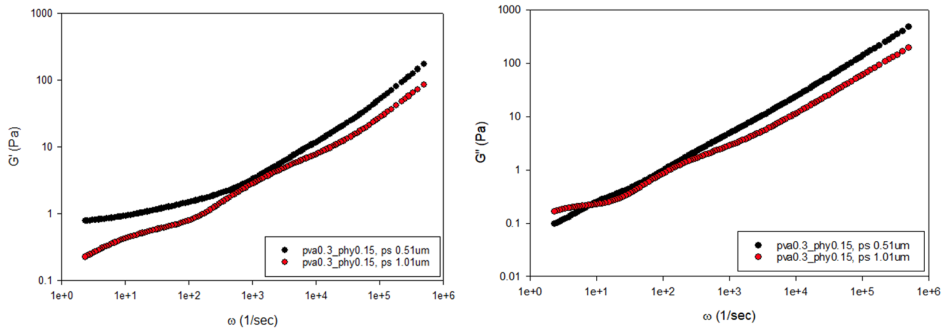

Figure S2. The elastic & viscous moduli read for Blend 3 PVA 0.30, PHY 0.15 with latex particles of varying size. At the lower and upper frequency limits, the non-overlapping results may be due to phase separation of the medium.

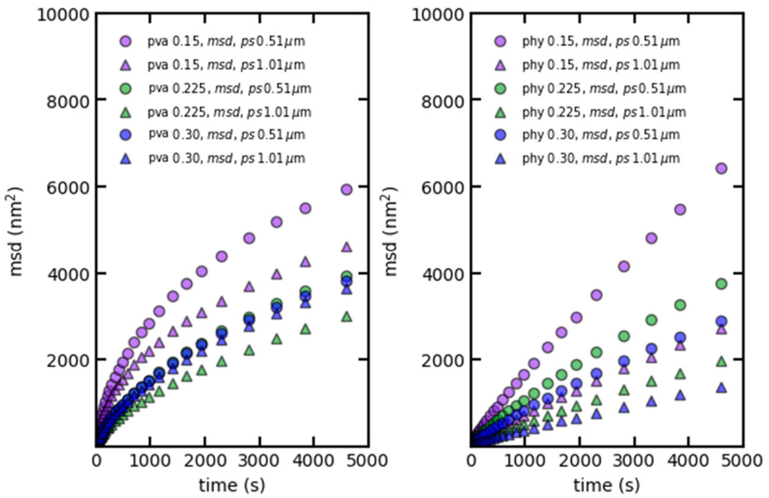

Figure S3. Confinement Effects in Poly(vinyl) alcohol and Phytagel: concentration-dependent mean square displacement analysis.

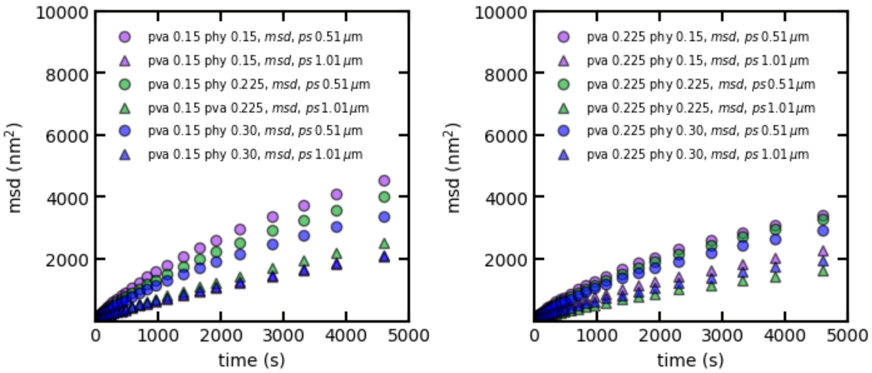

Figure S4. The mean square displacement of blends having different size probe particles showcasing confinement, as expected. The higher the concentration, the more confinement can be seen from the graph, as expected.

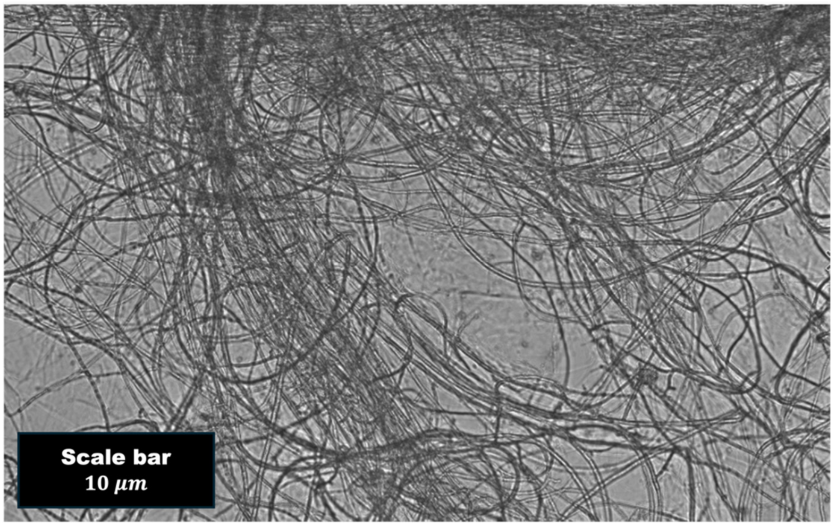

Figure S5. Microscopic Image of Sample (PVA 0.15, PHY 0.15% w/w) Captured with OLYMPUS IX51 Microscope. The polymer sample relates to physically entangled network.

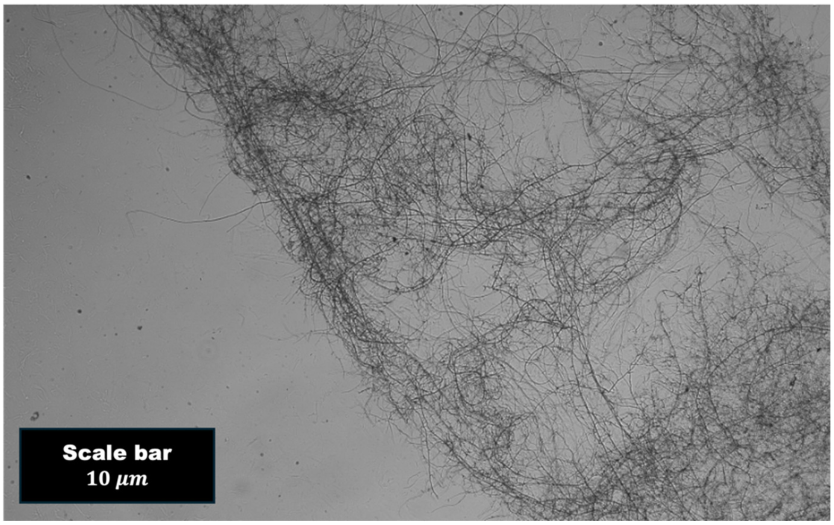

Figure S6. Microscopic Image of Sample (PVA 0.225, PHY 0.30% w/w) captured with OLYMPUS IX51 Microscope.

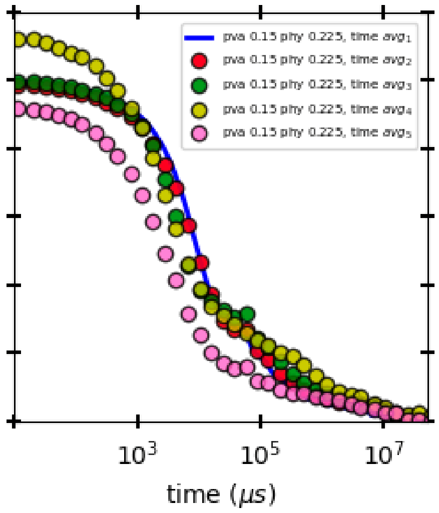

Figure S7. Auto-correlation function of Blend 2. Experimental Measurements at T = 25°C. Ensemble averaging conducted across various positions.

Table S2. Parameters for generalized Maxwell model for Phytagel of various concentrations with Polystyrene probe particles (ps) of size 0.51μm and 1.01μm embedded.

Sample	i	τ_i	G_i
**PHY 0.15 ps 0.51μm**	1	0.0340809	0.116486
	2	0.0115847	1.03E-07
	3	0.00393787	0.0761469
	4	0.00133856	0.44818
	5	0.000455	1.28515
	6	0.00015466	3.14116
	7	5.26E-05	7.21791
	8	1.79E-05	18.0656
	9	6.07E-06	38.9248
	10	2.06E-06	110.742
	11	7.02E-07	147.756
	12	2.39E-07	1.76E-05
	13	8.11E-08	8785.35
**PHY 0.15 ps 1.01μm**	1	0.00248888	0.394405
	2	0.00050101	1.85E+00
	3	0.00010085	7.91524
	4	2.03E-05	11.4884
	5	4.09E-06	2.12E-05
	6	8.23E-07	1.61E-08
	7	1.66E-07	10611.2
**PHY 0.225 ps 0.51μm**	1	0.320445	0.77142
	2	0.0473602	4.86E-10
	3	0.00699958	0.332754
	4	0.0010345	1.81523
	5	0.00015289	5.68033

	6	2.26E-05	15.5928
	7	3.34E-06	3.26E-08
	8	4.94E-07	2630
**PHY 0.225 ps 1.01μm**	1	0.00099959	1.07209
	2	0.00022203	4.0087
	3	4.93E-05	15.6922
	4	1.10E-05	43.0368
	5	2.43E-06	25.6968
	6	5.40E-07	2348.81
	7	1.20E-07	7.90E-08
	8	2.67E-08	2.50E-09
**PHY 0.30 ps 0.51μm**	1	0.00752279	0.250044
	2	0.00157894	0.483207
	3	0.000331401	4.04147
	4	6.96E-05	15.1603
	5	1.46E-05	48.5228
	6	3.06E-06	1.25E-07
	7	6.43E-07	1853.16
**PHY 0.30 ps 1.01μm**	1	0.0213076	0.377498
	2	0.00549154	1.00E-10
	3	0.00141532	1.01196
	4	0.00036477	3.70567
	5	9.40E-05	15.2191
	6	2.42E-05	26.7719
	7	6.24E-06	93.4583
	8	1.61E-06	3.13E-10
	9	4.15E-07	2945.86

Table S3. Parameters for generalized Maxwell model for Poly(vinyl) alcohol of various concentrations with Polystyrene probe particles (ps) of size 0.51μm and 1.01μm embedded.

Sample	i	τ_i	G_i
**PVA 0.15 ps 0.51μm**	1	0.418966	1.14E-09
	2	0.0712295	1.13E-09
	3	0.0121099	0.549696
	4	0.00205883	0.334283
	5	0.00035003	1.41514
	6	5.95E-05	2.57305
	7	1.01E-05	15.0016
	8	1.72E-06	7.39E-10
	9	2.92E-07	3.22E-07
	10	4.97E-08	1.15E-09
	11	8.45E-09	9.33E-08
	12	1.44E-09	292845
**PVA 0.15 ps 1.01μm**	1	0.418966	1.14E-09

	2	0.0712295	1.13E-09
	3	0.0121099	0.549696
	4	0.00205883	0.334283
	5	0.00035003	1.41514
	6	5.95E-05	2.57305
	7	1.01E-05	15.0016
	8	1.72E-06	7.39E-10
	9	2.92E-07	3.22E-07
	10	4.97E-08	1.15E-09
	11	8.45E-09	9.33E-08
	12	1.44E-09	292845
**PVA 0.225 ps 0.51μm**	1	0.4524	0.763788
	2	0.0488222	1.45E-08
	3	0.00526879	0.623445
	4	0.0005686	2.7942
	5	6.14E-05	13.6181
	6	6.62E-06	8.23E-08
	7	7.15E-07	1881.84
**PVA 0.225 ps 1.01μm**	1	0.689548	0.547209
	2	0.155331	2.58E-09
	3	0.0349907	0.0480141
	4	0.00788218	0.33215
	5	0.00177558	1.54318
	6	0.00039998	3.16152
	7	9.01E-05	6.29764
	8	2.03E-05	11.4427
	9	4.57E-06	1.13E-10
	10	1.03E-06	1116.41
**PVA 0.30 ps 0.51μm**	1	0.110275	1.0729
	2	0.0159097	1.24E-10
	3	0.00229536	0.683149
	4	0.00033116	4.34563
	5	4.78E-05	9.61424
	6	6.89E-06	3.54E-07
	7	9.94E-07	5.92E-05
	8	1.43E-07	0.0105128
	9	2.07E-08	1.05E-10
	10	2.99E-09	779636
**PVA 0.30 ps 1.01μm**	1	0.00896286	1.27707
	2	0.00161012	0.694813
	3	0.00028925	4.60334
	4	5.20E-05	7.60479
	5	9.33E-06	32.0998
	6	1.68E-06	180.064
	7	3.01E-07	0.00010078

	8	5.41E-08	6.63E-05
	9	9.72E-09	83939.4

Table S4. Parameters for generalized Maxwell model for Blend 1: Poly(vinyl) alcohol 0.15 %(w/w) and Phytagel 0.15-0.30 % (w/w) of various concentrations with Polystyrene probe particles (ps) of size 0.51μm and 1.01μm embedded.

Sample	i	τ_i	G_i
**PVA 0.225 PHY 0.15 ps 0.51μm**	1	0.012201	0.910197
	2	0.00291316	3.05E-07
	3	0.00069556	1.5354
	4	0.00016608	3.683
	5	3.97E-05	10.6255
	6	9.47E-06	6.22E-08
	7	2.26E-06	306.692
	8	5.40E-07	0.00130688
	9	1.29E-07	2.02E-10
	10	3.08E-08	0.00375798
	11	7.35E-09	2.86328
	12	1.75E-09	8.06749
	13	4.19E-10	140.393
	14	1.00E-10	8.22E+06
**PVA 0.15 PHY 0.15 ps 1.01μm**	1	0.0463705	0.56754
	2	0.0195753	5.02E-07
	3	0.00826374	1.45E-07
	4	0.00348854	3.67E-07
	5	0.00147269	0.536665
	6	0.0006217	1.24569
	7	0.00026245	2.94459
	8	0.00011079	3.56372
	9	4.68E-05	6.28144
	10	1.97E-05	13.691
	11	8.34E-06	26.8123
	12	3.52E-06	0.00023964
	13	1.49E-06	0.00181698
	14	6.27E-07	2194.5
**PVA 0.15 PHY 0.225 ps 0.51μm**	1	0.0176152	0.761079
	2	0.00355904	0.0572091
	3	0.00071908	1.74451
	4	0.00014529	5.70248
	5	2.94E-05	16.0682
	6	5.93E-06	84.5676
	7	1.20E-06	4.95E-10
	8	2.42E-07	0.00043171
	9	4.89E-08	2.96E-09
	10	9.88E-09	0.326494
	11	2.00E-09	4.85095

	12	4.03E-10	2.56E+06
**PVA 0.15 PHY 0.225 ps 1.01μm**	1	0.190536	9.42E-10
	2	0.0453851	0.437844
	3	0.0108106	3.11E-08
	4	0.00257506	0.114077
	5	0.00061337	2.89697
	6	0.0001461	5.26821
	7	3.48E-05	13.5435
	8	8.29E-06	42.789
	9	1.97E-06	1.01E-10
	10	4.70E-07	1829.23
**PVA 0.15 PHY 0.30 ps 0.51μm**	1	0.0430509	0.890639
	2	0.00536703	0.198522
	3	0.00066909	3.1759
	4	8.34E-05	13.0928
	5	1.04E-05	55.5186
	6	1.30E-06	281.462
	7	1.62E-07	5845.92
**PVA 0.15 PHY 0.30 ps 1.01μm**	1	0.0192243	0.459622
	2	0.00394938	0.246889
	3	0.00081135	2.12869
	4	0.00016668	5.31938
	5	3.42E-05	13.7554
	6	7.03E-06	42.9625
	7	1.45E-06	2.13E-05
	8	2.97E-07	3201.64

Table S5. Parameters for generalized Maxwell model for Blend 2: Poly(vinyl) alcohol 0.225 % (w/w) and Phytagel 0.15-0.30 % (w/w) of various concentrations with Polystyrene probe particles (ps) of size 0.51μm and 1.01μm embedded.

Sample	i	τ_i	G_i
**PVA 0.225 PHY 0.15 ps 0.51μm**	1	0.070685	0.97032
	2	0.0159012	4.45E-10
	3	0.00357711	0.199952
	4	0.0008047	1.68027
	5	0.00018103	5.22643
	6	4.07E-05	14.7122
	7	9.16E-06	31.8829
	8	2.06E-06	7.49E-06
	9	4.64E-07	2316.28
**PVA 0.225 PHY 0.15 ps 1.01μm**	1	0.281129	1.30E-07
	2	0.0715787	0.508974
	3	0.0182247	2.87E-08
	4	0.00464022	0.128746
	5	0.00118145	1.52765

	6	0.00030081	3.35199
	7	7.66E-05	4.68449
	8	1.95E-05	19.2336
	9	4.97E-06	1.35E-07
	10	1.26E-06	7.33E-06
	11	3.22E-07	2175.65
**PVA 0.225 PHY 0.225 ps 0.51μm**	1	5.46663	0.637736
	2	0.744232	7.20E-10
	3	0.10132	0.139153
	4	0.0137939	0.206771
	5	0.00187791	1.05106
	6	0.00025566	5.41823
	7	3.48E-05	14.034
	8	4.74E-06	8.85E-05
	9	6.45E-07	3145.49
**PVA 0.225 PHY 0.225 ps 1.01μm**	1	12.4535	0.354617
	2	1.5069	4.61E-09
	3	0.182338	0.139486
	4	0.0220633	0.182162
	5	0.00266971	0.758829
	6	0.00032304	5.64257
	7	3.91E-05	43.4656
	8	4.73E-06	2.57E-08
	9	5.72E-07	5.45E-05
	10	6.93E-08	10.0416
	11	8.38E-09	2.73E-05
	12	1.01E-09	7.29E-05
	13	1.23E-10	9.38E+06
**PVA 0.225 PHY 0.30 ps 0.51μm**	1	15.2413	0.715242
	2	2.93933	3.78E-08
	3	0.566861	0.0340724
	4	0.109321	0.10878
	5	0.0210829	0.153299
	6	0.00406592	0.305839
	7	0.00078413	2.26789
	8	0.00015122	7.22297
	9	2.92E-05	24.6431
	10	5.62E-06	3.71E-07
	11	1.08E-06	1446.36
**PVA 0.225 PHY 0.30 ps 1.01μm**	1	19.0479	0.280918
	2	4.56868	2.92E-08
	3	1.09581	0.0256205
	4	0.262832	0.118416
	5	0.063041	0.0836427
	6	0.0151205	1.20E-07

7	0.00362669	0.401496
8	0.00086987	2.34036
9	0.00020864	5.70937
10	5.00E-05	12.7777
11	1.20E-05	5.73E-07
12	2.88E-06	435.216

Sample Uncertainty

Reported confidence interval $(ci_{high} - ci_{low})$ width (at frequency of $\omega =$ 8000 Hz

1. PHY 0.15, ps 0.51μm:
    - At ω = 80000 Hz, in our measurements, the storage modulus G' was found to be **35.32** Pa with a 68% confidence interval width.**1.94** Pa, resulting in an uncertainty of $\pm\mathbf{0.970}$ Pa around the mean.
    - At ω = 80000 Hz, in our measurements, the loss modulus G'' was found to be **92.18** Pa with a 68% confidence interval width **8.169** Pa, resulting in an uncertainty of $\pm\mathbf{4.084}$ Pa around the mean.
2. PHY 0.15, ps 1.01μm:
    - At ω = 8000 Hz, in our measurements, the storage modulus G' was found to be **51.71** Pa with a 68% confidence interval width.**4.687** Pa, resulting in an uncertainty of $\pm\mathbf{2.343}$ Pa around the mean.
    - At ω = 8000 Hz, in our measurements, the loss modulus G'' was found to be **92.18** Pa with a 68% confidence interval width **19.032** Pa, resulting in an uncertainty of $\pm\mathbf{9.516}$ Pa around the mean.
3. PHY 0.30, ps 0.51μm:
    - At ω = 8000 Hz, in our measurements, the storage modulus G' was found to be **53.07** Pa with a 68% confidence interval width **5.887** Pa, resulting in an uncertainty of $\pm\mathbf{2.943}$ Pa around the mean.
    - At ω = 8000 Hz, in our measurements, the loss modulus G'' was found to be **128.43** Pa with a 68% confidence interval width **3.365** Pa, resulting in an uncertainty of $\pm\mathbf{1.682}$ Pa around the mean.
4. PHY 0.30, ps 1.01μm:
    - At ω = 80000 Hz, in our measurements, the storage modulus G' was found to be **75.12** Pa with a 68% confidence interval width **7.916** Pa, resulting in an uncertainty of $\pm\mathbf{3.958}$ Pa around the mean.

- At ω = 80000 $\boldsymbol{Hz}$, in our measurements, the loss modulus $G^{"}$ was found to be **170.28** Pa with a 68% confidence interval width **5.288** Pa, resulting in an uncertainty of $\pm\mathbf{2.6444}$ Pa around the mean.

5. PVA 0.15, ps 0.51μm:
    - At ω = 80000 $\boldsymbol{Hz}$, in our measurements, the storage modulus G' was found to be **23.27** Pa with a 68% confidence interval width.**1.2513** Pa, resulting in an uncertainty of $\pm\mathbf{0.626}$ Pa around the mean.
    - At ω = 80000 $\boldsymbol{Hz}$, in our measurements, the loss modulus $G^{"}$ was found to be **62.79** Pa with a 68% confidence interval width **0.976** Pa, resulting in an uncertainty of $\pm\mathbf{0.488}$ Pa around the mean.
6. PVA 0.15, ps 1.01μm:
    - At ω = 8000 $\boldsymbol{Hz}$, in our measurements, the storage modulus G' was found to be **37.51** Pa with a 68% confidence interval width **0.631** Pa, resulting in an uncertainty of $\pm\mathbf{0.315}$ Pa around the mean.
    - At ω = 8000 $\boldsymbol{Hz}$, in our measurements, the loss modulus $G^{"}$ was found to be **105.18** Pa with a 68% confidence interval width **0.493** Pa, resulting in an uncertainty of $\pm\mathbf{0.246}$ Pa around the mean.

7. PVA 0.30, ps 0.51μm:
    - At ω = 8000 $\boldsymbol{Hz}$, in our measurements, the storage modulus G' was found to be **17.44** Pa with a 68% confidence interval width **5.592** Pa, resulting in an uncertainty of $\pm\mathbf{2.796}$ Pa around the mean.
    - At ω = 8000 $\boldsymbol{Hz}$, in our measurements, the loss modulus $G^{"}$ was found to be **64.99** Pa with a 68% confidence interval width **9.274** Pa, resulting in an uncertainty of $\pm\mathbf{4.637}$ Pa around the mean.
8. PVA 0.30, ps 1.01μm:
    - At ω = 80000 $\boldsymbol{Hz}$, in our measurements, the storage modulus G' was found to be **18.94** Pa with a 68% confidence interval width **2.204** Pa, resulting in an uncertainty of $\pm\mathbf{1.102}$ Pa around the mean.
    - At ω = 80000 $\boldsymbol{Hz}$, in our measurements, the loss modulus $G^{"}$ was found to be **76.18** Pa with a 68% confidence interval width **1.381** Pa, resulting in an uncertainty of $\pm\mathbf{0.690}$ Pa around the mean.

9. PVA 0.15, PHY 0.15, ps 0.51μm:
    - At ω = 80000 $\boldsymbol{Hz}$, in our measurements, the storage modulus G' was found to be **28.62** Pa with a 68% confidence interval width.**8.232** Pa, resulting in an uncertainty of $\pm\mathbf{4.116}$ Pa around the mean.
    - At ω = 80000 $\boldsymbol{Hz}$, in our measurements, the loss modulus $G^{"}$ was found to be **117.92** Pa with a 68% confidence interval width **43.758** Pa, resulting in an uncertainty of $\pm\mathbf{21.874}$ Pa around the mean.
10. PVA 0.15, PHY 0.15, ps 1.01μm:
    - At ω = 8000 $\boldsymbol{Hz}$, in our measurements, the storage modulus G' was found to be **41.30** Pa with a 68% confidence interval width **5.170** Pa, resulting in an uncertainty of $\pm\mathbf{2.585}$ Pa around the mean.
    - At ω = 8000 $\boldsymbol{Hz}$, in our measurements, the loss modulus $G^{"}$ was found to be **155.37** Pa with a 68% confidence interval width **42.314** Pa, resulting in an uncertainty of $\pm\mathbf{21.157}$ Pa around the mean.
11. PVA 0.15, PHY 0.30, ps 0.51μm:
    - At ω = 8000 $\boldsymbol{Hz}$, in our measurements, the storage modulus G' was found to be **43.25** Pa with a 68% confidence interval width **3.504** Pa, resulting in an uncertainty of $\pm\mathbf{1.752}$ Pa around the mean.
    - At ω = 8000 $\boldsymbol{Hz}$, in our measurements, the loss modulus $G^{"}$ was found to be **143.02** Pa with a 68% confidence interval width **10.423** Pa, resulting in an uncertainty of $\pm\mathbf{5.211}$ Pa around the mean.
12. PVA 0.15, PHY 0.30, ps 1.01μm:
    - At ω = 80000 $\boldsymbol{Hz}$, in our measurements, the storage modulus G' was found to be **30.83** Pa with a 68% confidence interval width **6.877** Pa, resulting in an uncertainty of $\pm\mathbf{3.438}$ Pa around the mean.
    - At ω = 80000 $\boldsymbol{Hz}$, in our measurements, the loss modulus $G^{"}$ was found to be **117.78** Pa with a 68% confidence interval width **20.668** Pa, resulting in an uncertainty of $\pm\mathbf{10.334}$ Pa around the mean.
13. PVA 0.225, PHY 0.15, ps 0.51μm:
    - At ω = 80000 $\boldsymbol{Hz}$, in our measurements, the storage modulus G' was found to be **41.72** Pa with a 68% confidence interval width.**2.311** Pa, resulting in an uncertainty of $\pm\mathbf{1.155}$ Pa around the mean.

- At ω = 80000 $\boldsymbol{Hz}$, in our measurements, the loss modulus $G^{"}$ was found to be **106.82** Pa with a 68% confidence interval width **8.551** Pa, resulting in an uncertainty of $\boldsymbol{\pm 4.275}$ Pa around the mean.

14. <u>PVA 0.225, PHY 0.15, ps 1.01μm</u>:
    - At ω = 8000 $\boldsymbol{Hz}$, in our measurements, the storage modulus G' was found to be **26.05** Pa with a 68% confidence interval width **4.060** Pa, resulting in an uncertainty of $\boldsymbol{\pm 2.030}$ Pa around the mean.
    - At ω = 8000 $\boldsymbol{Hz}$, in our measurements, the loss modulus $G^{"}$ was found to be **62.75** Pa with a 68% confidence interval width **3.735** Pa, resulting in an uncertainty of $\boldsymbol{\pm 1.867}$ Pa around the mean.
15. <u>PVA 0.225, PHY 0.30, ps 0.51μm</u>:
    - At ω = 8000 $\boldsymbol{Hz}$, in our measurements, the storage modulus G' was found to be **47.02** Pa with a 68% confidence interval width **2.921** Pa, resulting in an uncertainty of $\boldsymbol{\pm 1.460}$ Pa around the mean.
    - At ω = 8000 $\boldsymbol{Hz}$, in our measurements, the loss modulus $G^{"}$ was found to be **130.54** Pa with a 68% confidence interval width **0.123** Pa, resulting in an uncertainty of $\boldsymbol{\pm 0.061}$ Pa around the mean.
16. <u>PVA 0.225, PHY 0.30, ps 1.01μm</u>:
    - At ω = 80000 $\boldsymbol{Hz}$, in our measurements, the storage modulus G' was found to be **38.41** Pa with a 68% confidence interval width **17.896** Pa, resulting in an uncertainty of $\boldsymbol{\pm 8.948}$ Pa around the mean.
    - At ω = 80000 $\boldsymbol{Hz}$, in our measurements, the loss modulus $G^{"}$ was found to be **91.74** Pa with a 68% confidence interval width **0.123** Pa, resulting in an uncertainty of $\boldsymbol{\pm 0.0618}$ Pa around the mean.

Table S6. Insights on the distribution of data for samples with repetition of 3, n=3 at ω = 8,0000 Hz.

Sample		Mean (Pa)		CI Width		Uncertainty	
		G'	$G^{"}$	G'	$G^{"}$	G'	$G^{"}$
1	PHY 0.15, ps 0.51μm	**35.32**	**92.18**	**1.94**	**8.169**	$\boldsymbol{\pm 0.970}$	$\boldsymbol{\pm 4.084}$
2	PHY 0.15, ps 1.01μm	**51.71**	**92.18**	**4.68**	**19.032**	$\boldsymbol{\pm 2.34}$	$\boldsymbol{\pm 9.516}$
3	PHY 0.30, ps 0.51μm	**53.07**	**128.43**	**5.887**	**3.365**	$\boldsymbol{\pm 2.943}$	$\boldsymbol{\pm 1.682}$
4	PHY 0.30, ps 1.01μm	**75.12**	**170.28**	**7.916**	**5.288**	$\boldsymbol{\pm 3.958}$	$\boldsymbol{\pm 2.6444}$
5	PVA 0.15, ps 0.51μm	**23.27**	**62.79**	**1.2513**	**0.976**	$\boldsymbol{\pm 0.626}$	$\boldsymbol{\pm 0.488}$

6	PVA 0.15, ps 1.01μm	37.51	105.18	0.631	0.493	± 0.315	± 0.246
7	PVA 0.30, ps 0.51μm	17.44	64.99	5.592	9.274	± 2.796	± 4.637
8	PVA 0.30, ps 1.01μm	18.94	76.18	2.204	1.381	± 1.102	± 0.690
9	PVA 0.15, PHY 0.15, ps 0.51μm	28.62	117.92	8.232	43.758	± 4.116	± 21.874
10	PVA 0.15, PHY 0.15, ps 1.01μm	41.30	155.37	5.170	42.314	± 2.585	± 21.157
11	PVA 0.15, PHY 0.30, ps 0.51μm	43.25	143.02	3.504	10.423	± 1.752	± 5.211
12	PVA 0.15, PHY 0.30, ps 1.01μm	30.83	117.78	6.877	20.668	± 3.438	± 10.334
13	PVA 0.225, PHY 0.15, ps 0.51μm	41.72	106.82	1.155	8.551	± 1.155	± 4.275
14	PVA 0.225, PHY 0.15, ps 1.01μm	26.05	62.75	4.060	3.735	± 2.030	± 1.867
15	PVA 0.225, PHY 0.30, ps 0.51μm	47.02	130.54	2.921	19.437	± 19.460	± 9.718
16	PVA 0.225, PHY 0.30, ps 1.01μm	38.41	91.74	17.896	21.669	± 8.948	± 10.834
